# Predicting disability and mortality in CV2/CRMP5‐IgG associated paraneoplastic neurologic disorders

**DOI:** 10.1002/acn3.51991

**Published:** 2024-01-22

**Authors:** Sanem P. Uysal, Yadi Li, Nicolas R. Thompson, Alex Milinovich, Justin R. Abbatemarco, Jeffrey A. Cohen, Devon S. Conway, Daniel Ontaneda, John A. Morren, Amy Kunchok

**Affiliations:** ^1^ Department of Neurology Neurological Institute, Cleveland Clinic Cleveland Ohio USA; ^2^ Department of Quantitative Health Sciences Lerner Research Institute, Cleveland Clinic Cleveland Ohio USA; ^3^ Center for Outcomes Research & Evaluation Neurological Institute, Cleveland Clinic Cleveland Ohio USA; ^4^ Mellen Center for Multiple Sclerosis Neurological Institute, Cleveland Clinic Cleveland Ohio USA; ^5^ Neuromuscular Center Neurological Institute, Cleveland Clinic Cleveland Ohio USA

## Abstract

**Background:**

We aimed to investigate the prognostic factors associated with clinical outcomes in CV2/Collapsin response‐mediator protein 5 (CRMP5)‐IgG paraneoplastic neurologic disorders (PND).

**Methods:**

This is a retrospective study of patients with CV2/CRMP5‐IgG PND evaluated between 2002–2022. We examined the association of clinical variables (including age, clinical phenotype [autoimmune encephalopathy, myelopathy, polyneuropathy/radiculopathy, MG, cerebellar ataxia, chorea, optic neuropathy], cancer) with three clinical outcomes (wheelchair dependence, modified Rankin Scale [mRS], mortality) using univariate logistic regression and Cox proportional hazards modeling. Kaplan–Meier estimates were used to determine the probability of survival.

**Results:**

Twenty‐seven patients (56% female) with CV2/CRMP5‐IgG PND were identified with a median follow‐up of 54 months (IQR = 11–102). An underlying tumor was identified in 15 patients (56%) including small cell lung cancer (SCLC) (8, [53%]), thymoma (4, [27%]), and other histologies (3, [20%]). At last follow‐up, 10 patients (37%) needed a wheelchair for mobility and this outcome was associated with myelopathy (HR = 7.57, 95% CI = 1.87–30.64, *P* = 0.005). Moderate–severe mRS = 3–5 was associated with CNS involvement (encephalopathy, myelopathy, or cerebellar ataxia) (OR = 7.00, 95% CI = 1.18–41.36, *P* = 0.032). The probability of survival 4 years after symptom onset was 66%. Among cancer subtypes, SCLC (HR = 18.18, 95% CI = 3.55–93.04, *P* < 0.001) was significantly associated with mortality, while thymoma was not.

**Interpretation:**

In this retrospective longitudinal study of CV2/CRMP5‐IgG PND, patients with CNS involvement, particularly myelopathy, had higher probability of disability. SCLC was the main determinant of survival in this population.

## Introduction

Paraneoplastic neurological disorders (PND) are rare, and there is limited literature on longitudinal outcomes in this population. Although rare, with increasing availability of diagnostic antibody biomarkers, improved awareness, cancer therapies (such as immune checkpoint inhibitors [ICI]), and improved cancer survival, patients with PND are increasingly cared for longitudinally in neurology outpatient clinics. One common issue faced by neurologists and oncologists caring for these patients is in prognosticating outcomes including disability and mortality.

Collapsin response‐mediator protein 5 (CRMP5) immunoglobulin G (IgG) (also known as anti‐CV2 antibody) is a paraneoplastic antibody first described in patients presenting with limbic encephalitis, myelitis, myasthenic syndrome, and various malignancies.^1^ CV2/CRMP5‐IgGs are not thought to be directly pathogenic, but more likely reflect paraneoplastic T‐cell driven neurological autoimmunity.^2^ Detection of CV2/CRMP5‐IgG is rare and accounts for <1% of neural autoantibodies found in patients tested for autoimmune encephalitis in large neuroimmunology laboratories.^3^

The most common malignancies associated with CV2/CRMP5‐IgG PND are small cell lung cancer (SCLC) and thymoma.^4^, ^5^ As is true with other PND, neoplasms associated with CV2/CRMP5‐IgG can be occult and the emergence of neurological symptoms may precede their detection by months or even years.^6^ Rarely, ICI's have been associated with CV2/CRMP5‐IgG PND.^7^

CV2/CRMP5‐IgG PND can have both central and peripheral nervous system manifestations, including myelopathy, limbic encephalitis, cranial neuropathies (most notably optic neuropathy), cerebellar ataxia, myasthenic syndromes, polyneuropathy, radiculopathy, and chorea.^6^, ^8^, ^9^, ^10^ It is not fully understood why some patients manifest certain neurological symptoms and others do not. However, tumor type may have some influence, as myasthenia gravis (MG) and Lambert Eaton syndrome (LEMS) are often associated with thymoma in CV2/CRMP5‐IgG PND.^11^, ^12^

Current standard practice of CV2/CRMP5‐IgG PND includes identification and management of the underlying tumor, and immunosuppressive treatment directed at the neurological symptoms.^6^, ^9^, ^10^ Given the rarity of CV2/CRMP5‐IgG PND, longitudinal outcomes are not yet well defined. It is unclear whether duration of the PND, the neurological manifestations (including central versus peripheral nervous system, or tumor type (SCLC versus thymoma) are associated with different clinical outcomes. The understanding of these factors may allow clinicians to prognosticate outcomes and may also impact management decisions.

In this study, we aimed to evaluate whether clinical factors predict outcomes including wheelchair dependence, global disability (as determined by modified Rankin Scale, [mRS]), and survival in patients with CV2/CRMP5‐IgG PND.

## Methods

### Study design and standard protocol approval

This was a retrospective study of patients with CV2/CRMP5‐IgG PND who were evaluated at the Cleveland Clinic between 2002 and 2022. The study was approved by the Cleveland Clinic Institutional Review Board, and participant consent was waived. The study population was derived from the autoimmune neurology registry at the Cleveland Clinic and from a search of the electronic medical record. All patients with serum and/or cerebrospinal fluid CV2/CRMP5‐IgG antibody positivity by western blot and/or tissue immunofluorescence and meeting PND criteria were evaluated for inclusion. Antibody testing was completed using Mayo Clinic Laboratories, Quest Diagnostics, or Athena Diagnostics. A detailed review of clinical features was undertaken to determine that included patients fulfilled the PND diagnostic criteria.^14^ Patients with fewer than <2 months of follow‐up were excluded (Figure S1).

### Prognostic variables

Demographic and clinical characteristics included age at symptom onset, sex, clinical phenotype (autoimmune encephalopathy, myelopathy, polyneuropathy/radiculopathy, MG, cerebellar ataxia, chorea, optic neuropathy), smoking history, presence of pulmonary nodules, presence of cancer and type, and immunosuppressive treatment. CNS involvement due to PND was defined as the presence of autoimmune encephalopathy, myelopathy, cerebellar ataxia, and/or chorea. All patients with MG were diagnosed based on serology (acetylcholine receptor [AChR‐IgG] positivity) and clinical evaluation by their neurologist. All patients with paraneoplastic polyneuropathy or radiculopathy were diagnosed if any of these EMG patterns were present: painful axonal polyneuropathy, asymmetric or symmetric polyradiculoneuropathy, sensory neuronopathy, autonomic or small fiber neuropathy and if no alternative etiology of neuropathy was identified. CSF was defined as inflammatory if any of the following features were present: pleocytosis (>5 cells/*μ*L), elevated protein (>45 mg/dL), increased IgG index (>0.61), and oligoclonal bands (>1 unique IgG bands in the CSF compared to the serum).

### Clinical outcomes

Three disability and survival outcome measures were evaluated: wheelchair dependence, modified Rankin Scale (mRS) score at last visit, and mortality. mRS was used as an outcome measure for global disability, and scores were dichotomized as 0–2 (mild) and 3–5 (moderate–severe). Wheelchair dependence was evaluated as an additional disability outcome, reflective of neurological (motor or coordination) impairment.

### Statistical analysis

Descriptive statistics summarized patient and clinical characteristics by median with interquartile range (IQR) for continuous variables and count with percentage for categorical variables. Single‐predictor Cox proportional hazard models were used for prognostic factors associated with wheelchair dependence and death. Univariate logistic regression models examined prognostic factors associated with moderate–severe mRS. Kaplan–Meier estimates were used to evaluate the effect of CNS involvement, cancer diagnosis, and cancer type on time to wheelchair dependence and death. Fisher's exact tests were used to examine the associations between clinical phenotype and cancer comorbidity. Statistical significance was set at 0.05. Due to the exploratory nature of the study, we did not correct for multiple testing. Statistical tests were not performed for the analysis of the characteristics of the study population stratified by clinical phenotypes, as the phenotypes were not mutually exclusive. Analyses were conducted in SAS Enterprise Guide, version 8.2 (SAS Institute Inc, Cary, NC, USA).

## Results

### Study population: Baseline demographic and clinical features

There were 32 patients with CV2/CRMP5‐IgG positivity, 4 were excluded due to short follow‐up (<2 months), and 1 was excluded due to alternative diagnosis (progressive multifocal leukoencephalopathy) (Figure S1). A total of 27 patients were included (56% female, 96% white), with a median age at symptom onset of 65.0 years (IQR = 54–71). Median follow‐up time from symptom onset was 4.5 years (IQR = 0.9–8.3 years) (Table 1).

**Table 1:** Characteristics of the study sample.

	Total (*N* = 27)
Age at symptom onset (median [Q1, Q3], years)	65.0 [54.0, 71.0]
Male sex (*n*, %)	12/27 (44.4)
White ethnicity (*n*, %)	26/27 (96.3)
Follow‐up time from symptom onset (median [Q1, Q3], months)	53.9 [11.2, 102.2]
Cancer (*n*, %)	15/27 (55.6)
SCLC (*n*, %)	8/15 (53.3)
Thymoma (*n*, %)	4/15 (26.7)
Other (*n*, %)	3/15 (20.0)
Without cancer (*n*, %)	12/27 (44.4)
Pulmonary nodules^1^ (*n*, %)	7/12 (58.3)
Smoking history (*n*, %)	22/27 (81.5)
CSF with inflammation^1^ (*n*, %)	9/11 (81.8)
Deceased (*n*, %)	13/27 (48.1)
Due to cancer (*n*, %)	5/13 (38.5)
Due to neurologic cause (*n*, %)	1/13 (7.7)
Other causes (*n*, %)	7/13 (53.8)
Received immunotherapy (*n*, %)	15/27 (55.6)
Acute immunotherapy (IVMP, IVIG or PLEX) (*n*, %)	11/15 (73.3)
Cyclophosphamide (*n*, %)	3/15 (20.0)
Mycophenolate mofetil (*n*, %)	1/15 (6.7)
Other (*n*, %)	4/15 (26.7)
Wheelchair dependence due to PND (*n*, %)	10/27 (37.0)
mRS at first visit (median [Q1, Q3])	2.0 [2.0, 3.0]
Moderate–severe mRS at first visit (mRS 3–5) (*n*, %)	11/27 (40.7)
mRS at last visit (median [Q1, Q3])	3.0 [2.0, 4.0]
Moderate–severe mRS at last visit (mRS 3–5) (*n*, %)	18/27 (66.7)
Clinical phenotypes	
Cerebellar ataxia (*n*, %)	11/27 (40.7)
Myelopathy (*n*, %)	8/27 (29.6)
Polyneuropathy/radiculopathy (*n*, %)	14/27 (51.9)
Myasthenia (*n*, %)	7/27 (25.9)
Autoimmune encephalopathy (*n*, %)	7/27 (25.9)
Optic neuropathy (*n*, %)	6/27 (22.2)

Statistics presented as median [P25, P75], *N* (column %). Abbreviations: SCLC, small cell lung cancer; CSF, cerebrospinal fluid; MRI, magnetic resonance imaging; IVMP, intravenous methylprednisolone; IVIG, intravenous immunoglobulin; PLEX, plasma exchange; mRS, modified Rankin Scale.

^1^ Data are not available for all subjects. Missing values: Pulmonary nodules = 4; CSF = 16.

**Figure 1:**
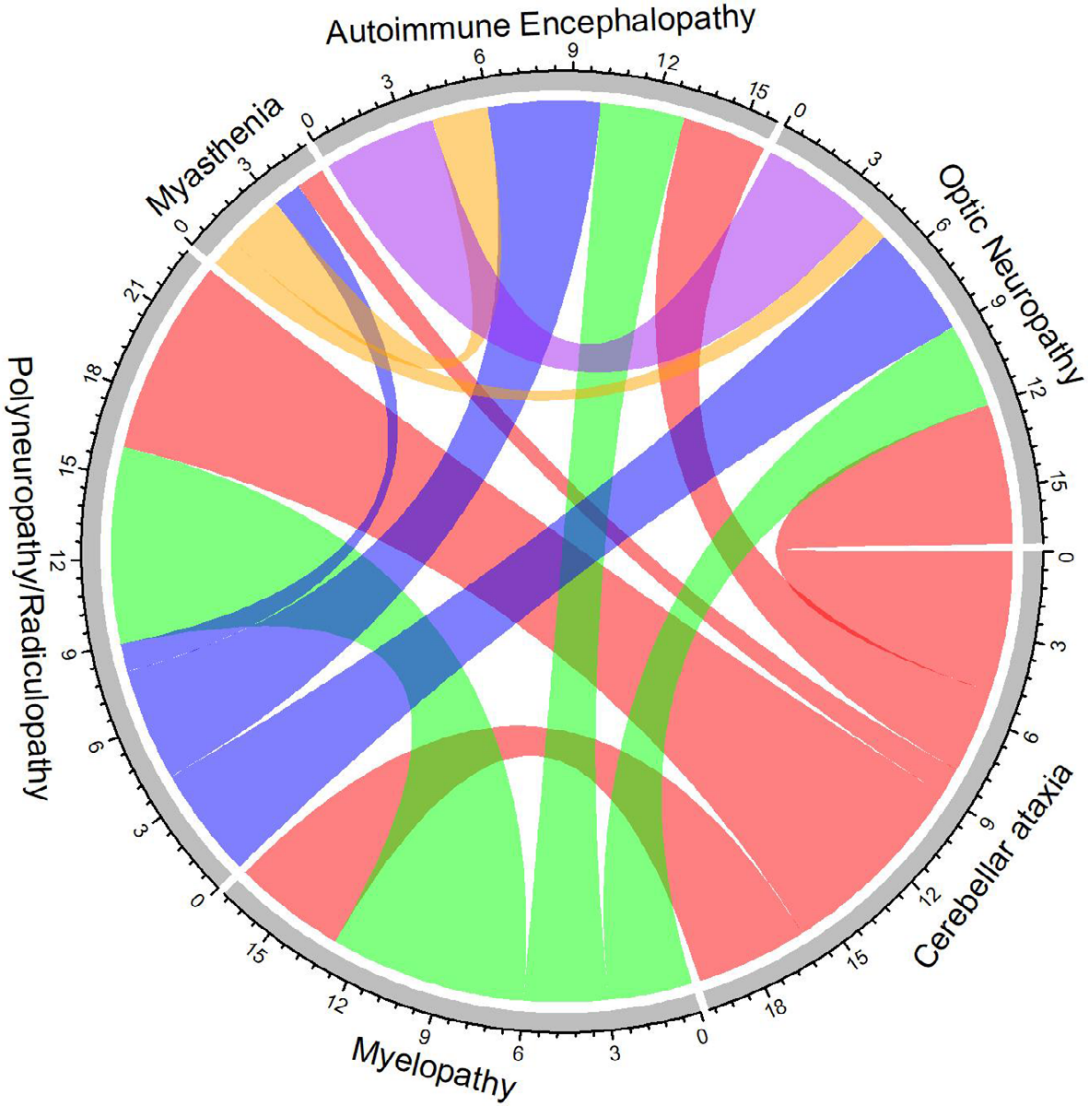
Chord diagram showing overlapping neurologic phenotypes. The size of the arc connecting two phenotypes indicates the number of patients who had both phenotypes.

Clinical phenotypes included polyneuropathy/radiculopathy (14, [52%]), cerebellar ataxia (11, [41%]), myelopathy (8, [30%]), MG (7, [26%]), autoimmune encephalopathy (7, [26%]), and optic neuropathy (6, [22%]) (Fig. 1, Figure S2, Table S1). Several patients had multiple clinical phenotypes. There were no patients with chorea in this cohort.

Cancers were identified in 15 (56%), including SCLC (8, [53%]), thymoma (4 [27%]), non‐small cell lung cancer (1 [6.7%]), renal cell carcinoma (1 [6.7%]), and neuroendocrine tumor (1 [6.7%]) (Figure S3). Patients with thymoma had a younger age at CV2/CRMP5‐IgG PND symptom onset (median = 43.0, IQR = 42.0–49.5) than non‐thymoma patients (*P* = 0.01). MG was associated with thymoma (*P* = 0.002) (Table S2).

Twenty‐two patients were smokers (82%) (Table 1). Of the 12 patients without cancer, 7 (58%) had pulmonary nodules and 9 (75%) were smokers. Only 3/12 (25%) had FDG‐PET body imaging for malignancy evaluation, significantly fewer than those with cancer (p = 0.016), suggesting a potential difference in cancer screening between the groups. Among the 15 patients with cancer, onset of neurologic symptoms preceded the cancer diagnosis in 11 patients (73%) by a median of 10 months (IQR: 6–12). The longest delay to cancer diagnosis was 48 months.

Two patients were treated with ICIs. One patient received nivolumab for metastatic renal cell cancer, with PND preceding ICI initiation and worsening neurologic status following ICI with development of cerebellar ataxia, optic neuropathy, myasthenic syndrome, polyneuropathy and autoimmune encephalopathy and died of respiratory failure. The second patient who received atezolizumab for metastatic small cell lung cancer developed myelopathy 3 months later and died of progression of cancer.

Of 7 patients with encephalopathy, brain MRI was abnormal in 2 patients (T2 lesions in 1 [50%], contrast‐enhancement in 1 [50%]). Of 8 patients with myelopathy, spine MRI was abnormal in 3 patients (T2 lesions in 1 [33%], contrast‐enhancement in 2 [67%]). CSF was inflammatory in 80% of tested patients (8/10). Immunosuppressive treatment, including IV steroids, IVIG, plasmapheresis, and steroid‐sparing agents, was given to 15 patients (56%) (Table 1).

### Prognostic factors associated with wheelchair dependence

**Figure 2:**
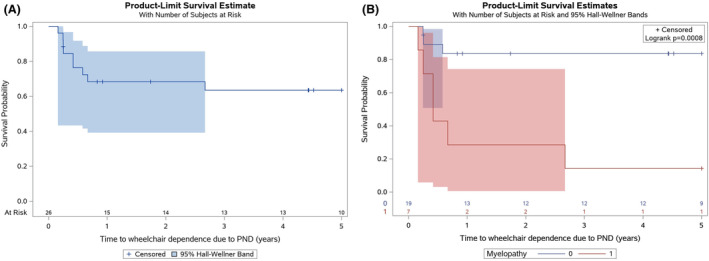
(A) Kaplan–Meier curve for time to wheelchair dependence due to paraneoplastic neurologic disorder. (B) Kaplan–Meier curve for time to wheelchair dependence due to paraneoplastic neurologic disorder, stratified by myelopathy (yes/no).

No patients were wheelchair‐dependent at the time of diagnosis of CV2/CRMP5‐IgG PND. Ten patients (37%) were wheelchair‐dependent at last follow‐up. Wheelchair dependence was associated with myelopathy (HR = 7.57, 95% CI = 1.87–30.64, *P* = 0.005) (Table 2). Other clinical phenotypes (cerebellar ataxia, polyneuropathy/radiculopathy, MG, encephalopathy, or optic neuropathy) were not associated with wheelchair dependence. Probability of wheelchair dependence due to PND at 4 years was 37% in the overall study population and 86% in those with myelopathy (Fig. 2A, B). Wheelchair dependence was also found to be associated with SCLC (HR = 4.68, 95% CI = 1.21–18.07, *P* = 0.025).

**Table 2:** Results of single‐predictor Cox proportional hazard models for wheelchair dependence.

	Wheelchair dependence	
	HR (95% CI)	*P*‐value
Age at symptom onset	1.08 (1.00–1.17)	0.050
Female	3.42 (0.71–16.56)	0.13
Cancer	3.42 (0.71–16.56)	0.13
SCLC	4.68 (1.21–18.07)	**0.025**
CNS	6.12 (0.76–49.02)	0.088
Cerebellar ataxia	1.45 (0.39–5.43)	0.58
Myelopathy	7.57 (1.87–30.64)	**0.005**
Autoimmune encephalopathy	1.01 (0.21–4.85)	0.99
Neuropathy/radiculopathy	2.20 (0.55–8.86)	0.27
Optic neuropathy	0.45 (0.06–3.61)	0.45

Bolded values indicate significant *P*‐value (<0.05). Abbreviations: SCLC, small cell lung cancer; CNS, central nervous system; HR, hazard ratio; CI, confidence interval.

### Prognostic factors associated with moderate–severe mRS (3–5)

**Figure 3:**
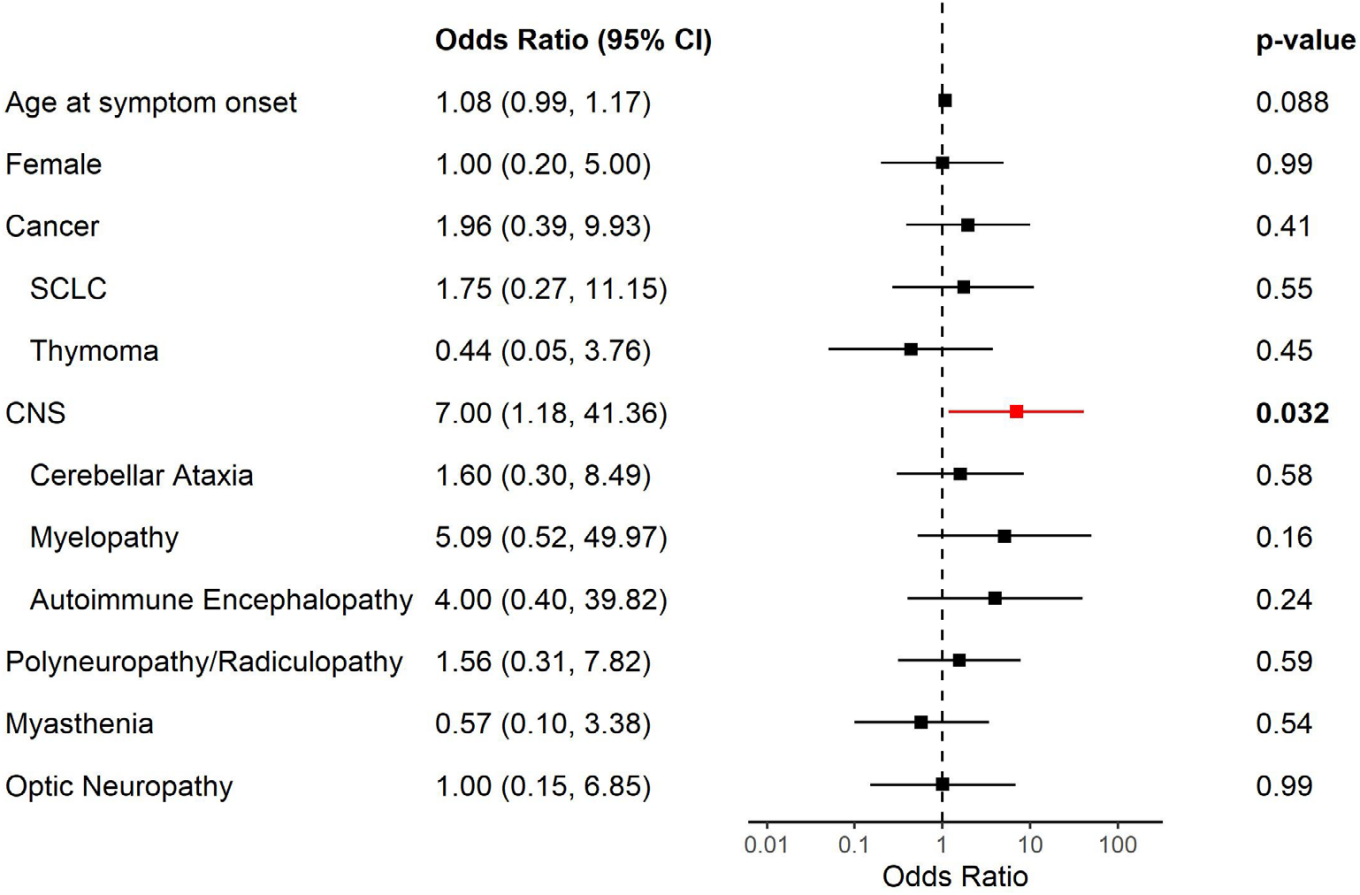
Forest plot with logistic regression predicting moderate–severe modified Rankin Score at last visit. Error bars: 95% confidence interval. SCLC, small cell lung cancer; CNS, central nervous system (encephalopathy, myelopathy or cerebellar ataxia).

The median mRS at baseline for the study population was 2 (IQR = 2–3), and 18 patients (67%) had mRS: 3–5 at last follow‐up. CNS involvement (cerebellar ataxia, encephalopathy or myelopathy) (OR = 6.50, CI = 1.09–38.63, *P* = 0.04) was associated with mRS 3–5 at last visit (Fig. 3). Patients with any CNS phenotypes were more likely to have moderate–severe mRS than patients with only peripheral nervous system (PNS) involvement. (Fig. 3 and Table S1).

### Prognostic factors associated with survival

**Figure 4:**
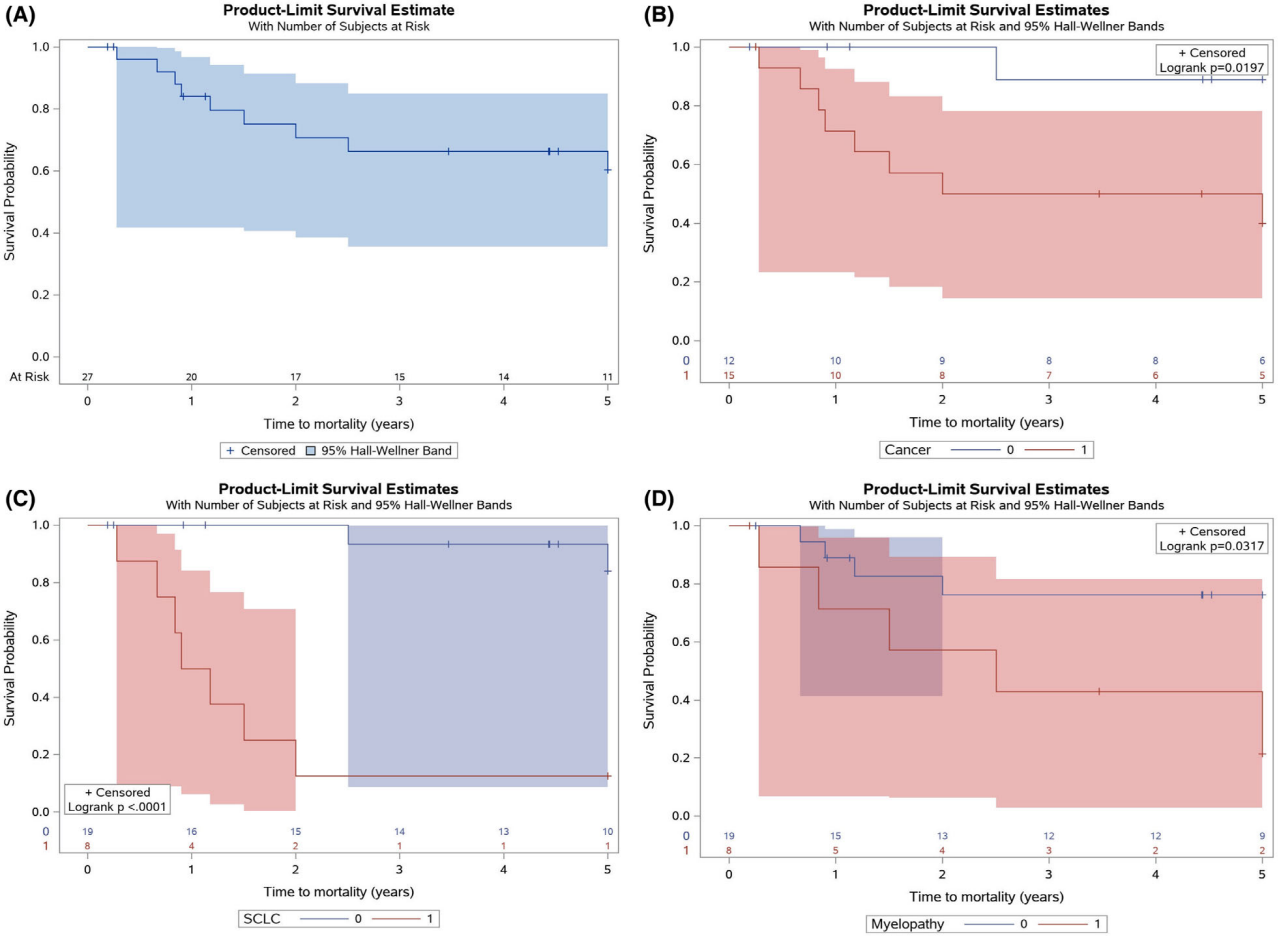
(A) Kaplan–Meier curve for time to mortality. (B) Kaplan–Meier curve for time to mortality, stratified by cancer (yes/no). (C) Kaplan–Meier curve for time to mortality, stratified by small cell lung cancer (yes/no). (D) Kaplan–Meier curve for time to mortality, stratified by myelopathy (yes/no).

The median survival after symptom onset for the study population was 17.7 months (IQR = 10.2–55.6). The probability of survival at 4 years after symptom onset was 66% (Fig. 4A). Age at onset was not associated with survival.

Predictors of mortality included SCLC (HR = 18.18, 95% CI = 3.55–93.04, *P* < 0.001) and myelopathy (HR = 15.17, 95% CI = 1.51–152.46, *P* = 0.021). Thymoma was not associated with mortality (Table 3, Table S2). Probability of survival at 4 years with cancer diagnosis was 50% compared to 88.9% without cancer diagnosis (Fig. 4B). Probability of survival at 4 years with SCLC was 12.5%. (Fig. 4C). Patients with myelopathy had a lower probability of survival at 4 years compared to those without myelopathy, based on Kaplan–Meier estimates (*P* = 0.032) (Fig. 4D).

**Table 3:** Results of single‐predictor Cox proportional hazard models for mortality.

	Mortality	
	HR (95% CI)	*P*‐value
Age at symptom onset	1.07 (0.99–1.15)	0.091
Female	8.03 (1.00–64.37)	0.050
Cancer	8.03 (1.00–64.37)	0.050
SCLC	18.18 (3.55–93.04)	**<0.001**
CNS	2.71 (0.56–13.16)	0.22
Cerebellar ataxia	0.51 (0.11–2.47)	0.40
Myelopathy	3.83 (1.03–14.33)	**0.046**
Autoimmune encephalopathy	2.05 (0.51–8.29)	0.32
Neuropathy/radiculopathy	2.16 (0.54–8.71)	0.28
Optic neuropathy	0.48 (0.06–3.83)	0.49

Bolded values indicate significant *P*‐value (<0.05). Abbreviations: SCLC, small cell lung cancer; CNS, central nervous system; HR, hazard ratio; CI, confidence interval.

Kaplan–Meier analyses were also performed to compare the survival of patients with cancer (15), with pulmonary nodules (7) and with neither (5). Probability of survival at 4 years was 57% in patients with either pulmonary nodules or a histological diagnosis of cancer. Probability of survival at 4 years was 100% in patients without cancer nor pulmonary nodules (Figures S4 and S5).

### Sensitivity analysis: prognostic factors in CV2/CRMP‐IgG PND without MG

The outcomes were re‐evaluated in statistical models with the 7 AChR‐IgG positive myasthenia patients removed. The results of the subgroup analyses remained similar (Table S3).

## Discussion

In this retrospective longitudinal study of CV2/CRMP5‐IgG PND, we identified several key factors associated with clinical outcomes. Firstly, paraneoplastic myelopathy and SCLC were associated with wheelchair dependence. Secondly, moderate–severe disability at last visit (mRS 3–5) was associated with CNS involvement (myelopathy, encephalopathy, or cerebellar ataxia). Finally, myelopathy and SCLC were also significant prognostic factors for mortality.

Among the CV2/CRMP5‐IgG PND clinical phenotypes, myelopathy was the only phenotype associated with wheelchair dependence. In contrast, in patients without myelopathy (e.g., polyneuropathy and MG), the probability of wheelchair dependence remained <20% at 4 years. These results are consistent with the results of a previous study showing a higher proportion of a progressive course in patients with CV2/CRMP5‐IgG myelopathy, which is a likely contributor to the increased disability associated with this clinical phenotype.^10^ Any CNS involvement (encephalopathy, ataxia, or myelopathy) was associated with moderate–severe mRS at last visit, highlighting the disability burden of paraneoplastic CNS disease compared to isolated PNS involvement.

In this patient population, the overall probability of survival at 4 years was 66%, which is comparable to that reported in a previous study.^12^ As expected, the presence of SCLC was strongly associated with mortality with a survival of 12.5% at 4 years. Conversely, thymoma was not associated with mortality. Patients with thymoma were younger and predominantly had MG as their clinical presentation of CV2/CRMP5‐IgG PND. These factors highlight that the type of cancer is a key driver of mortality in CV2/CRMP5‐IgG PND. Myelopathy was associated with wheelchair dependence, as expected, highlighting the significant disability experienced by patients with paraneoplastic myelopathy. SCLC was also associated with wheelchair dependence. This association may reflect the greater frequency of SCLC in patients with myelopathy or cancer morbidity in patients with SCLC.

We were underpowered to examine the subgroup with MG and thymoma alone and removal of these patients did not majorly change the findings of the pool cohort in terms of the prognostic factors. However, it is likely that patients with MG and thymoma represent a distinct clinical subgroup among the CV2/CRMP5‐IgG PND, with an overall better prognosis compared to those with SCLC and/or CNS manifestations. These findings highlight that clinical outcomes in patients with CV2/CRMP5‐IgG PND are driven by the underlying cancer and presence of CNS involvement.^13^

Although CV2/CRMP5‐IgG is a high‐risk paraneoplastic antibody, 44% of our patients did not have cancer identified at the time of the CV2/CRMP5‐IgG detection. However, these patients all had subacute neurological symptoms consistent with PND. Furthermore, this group had a high proportion of high‐risk features including pulmonary nodules and smoking history. We suspect that some of these patients may have been under‐evaluated for malignancy, with 75% of those without a cancer diagnosis not undergoing an FDG‐PET body scan. Additionally, it is possible that in some patients, the paraneoplastic process inhibits the macroscopic tumor progression.^14^, ^15^, ^16^ Similar findings were reported in a study evaluating patients with Hu/ANNA1‐IgG PND, which showed that 4% of patients had a cancer diagnosis more than 2 years after the onset of PND and only 10% of patients remained cancer‐free for more than 2 years, leading to a recommendation to screen early with PET scan and to extend overall cancer screening to 5 years.^17^

There were two patients within the cohort who received ICI's for cancer treatment. One patient developed PND prior to ICI initiation and worsened in neurological status after. The other patient developed PND 3 months following the last dose of ICI. These two novel cases (not previously reported) suggest that ICI treatment can enhance the immune response and facilitate or augment paraneoplastic neurologic symptoms, as reported in the literature.^7^, ^18^ Both patients died, consistent with other reports of higher disability and mortality in patients with PND and ICI exposure^19^.

This study has several strengths, including the availability of detailed clinical data and longitudinal follow‐up time of over 4 years for this rare paraneoplastic disorder. The retrospective observational design made testing and follow‐up variable; however, this reflects real world clinical practice. The risk of type 1 error could not be excluded due to inability to conduct multivariable testing owing to the rarity of the disorder and the exploratory nature of the analyses. The effect of immunosuppressive therapies could not be determined due to the heterogeneity of treatment types and the small sample size.

The findings from this study suggest that although these patients share a common PND antibody biomarker (CV2/CRMP5‐IgG), outcomes are heterogenous (as are the clinical phenotypes) and driven by cancer type and CNS involvement. These findings may help clinicians who care for patients with CV2/CRMP5‐IgG PND with prognosticating outcomes of disability and mortality.

## Author Contributions

Each named author has substantially contributed to conducting the research and drafting this manuscript.

## Potential Conflicts of Interest

Dr. Sanem Pinar Uysal has no disclosures. Yadi Li has no disclosures. Nicholas R. Thompson has no disclosures. Alex Milinovich has received research support from Merck, Pfizer, Twin Health, NFLPA & National Institute on Aging. Dr. Justin R. Abbatemarco has served on scientific advisory boards for EMD Serono, Genentech, and Horizon; has received research support from Horizon. Dr. Jeffrey Cohen discloses personal compensation for consulting for Astoria, Bristol‐Myers Squibb, Convelo, EMD Serono, FiND Therapeutics, INMune, and Sandoz; and serving as an Editor of Multiple Sclerosis Journal. Dr. Devon S. Conway has received research support paid to his institution from BMS, Novartis, EMD Serono, Biogen, Horizon Therapeutics and the National Institutes of Health. He has received consulting fees from Novartis and Alexion Pharmaceuticals and speaking fees from Biogen. Dr. Daniel Ontaneda discloses Research support from the National Institutes of Health, National Multiple Sclerosis Society, Patient Centered Outcomes Research Institute, Race to Erase MS Foundation, Genentech, Genzyme, and Novartis. Consulting fees from Biogen Idec, Bristol‐Myers Squibb, Genentech/Roche, Genzyme, Janssen, Novartis, and Merck. Dr. John A. Morren has no relevant disclosures to declare. Dr Amy Kunchok discloses personal compensation for consulting for Genentech, Horizon therapeutics, and EMD Serono.

## Supporting information


**Figure S1.** The flow chart of inclusion criteria of the study.

**Figure S2.** Upset plot for the frequency of neurologic phenotype.

**Figure S3.** Number of patients in each cancer type.

**Figure S4.** Kaplan–Meier curve for time to mortality, stratified by cancer/pulmonary nodules vs none.

**Figure S5.** Kaplan–Meier curve for time to mortality, stratified by cancer vs no cancer, with pulmonary nodules vs none.

**Table S1.** Characteristics of the study sample, stratified by phenotypes.
**Table S2.** Comparison between patients with and without thymoma.
**Table S3.** Comparison between patients with and without myasthenia gravis.

## Data Availability

Anonymized data not published within this article including raw datasets, analysis‐ready datasets, and statistical analysis plan may be shared based on reasonable request by qualified investigators for purposes of replicating procedures and results.

## References

[1] Honnorat J , Antoine JC , Derrington E , Aguera M , Belin MF . Antibodies to a subpopulation of glial cells and a 66 kDa developmental protein in patients with paraneoplastic neurological syndromes. J Neurol Neurosurg Psychiatry. 1996;61(3):270‐278. doi:10.1136/JNNP.61.3.270 8795598 PMC486550

[2] Antoine JC , Honnorat J , Camdessanché JP , et al. Paraneoplastic anti‐CV2 antibodies react with peripheral nerve and are associated with a mixed axonal and demyelinating peripheral neuropathy. Ann Neurol. 2001;49:214‐221. 11220741 10.1002/1531-8249(20010201)49:2<214::aid-ana41>3.0.co;2-w

[3] Kunchok A , McKeon A , Zekeridou A , et al. Autoimmune/paraneoplastic encephalitis antibody biomarkers: frequency, age, and sex associations. Mayo Clin Proc. 2022;97(3):547‐559. doi:10.1016/j.mayocp.2021.07.023 34955239

[4] Rogemond V , Honnorat J . Anti‐CV2 autoantibodies and paraneoplastic neurological syndromes. Clin Rev Allergy Immunol. 2000;19:51‐60. 11064826 10.1385/CRIAI:19:1:51

[5] Yu Z , Kryzer TJ , Griesmann GE , Kim KK , Benarroch EE , Lennon VA . CRMP‐5 neuronal autoantibody: marker of lung cancer and thymoma‐related autoimmunity. Ann Neurol. 2001;49(2):146‐154. doi:10.1002/1531-8249(20010201)49:2<146::AID-ANA34>3.0.CO;2-E 11220734

[6] Dubey D , Lennon VA , Gadoth A , et al. Autoimmune CRMP5 neuropathy phenotype and outcome defined from 105 cases. Neurology. 2018;90(2):e103‐e110. doi:10.1212/WNL.0000000000004803 29222126

[7] Kunchok A , Zekeridou A , Pittock S . CRMP5‐IgG‐associated paraneoplastic myelopathy with PD‐L1 inhibitor therapy. JAMA Neurol. 2020;77(2):255‐256. doi:10.1001/jamaneurol.2019.4379 31860013

[8] Cohen DA , Bhatti MT , Pulido JS , et al. Collapsin response‐mediator protein 5–associated retinitis, vitritis, and optic disc edema. Ophthalmology. Vol 127. Elsevier Inc; 2020:221‐229. doi:10.1016/j.ophtha.2019.09.012 31676123

[9] Cross SA , Salomao DR , Parisi JE , et al. Paraneoplastic autoimmune optic neuritis with retinitis defined by CRMP‐5‐IgG. Ann Neurol. 2003;54(1):38‐50. doi:10.1002/ana.10587 12838519

[10] Keegan BM , Pittock SJ , Lennon VA . Autoimmune myelopathy associated with collapsin response‐mediator protein‐5 immunoglobulin G. Ann Neurol. 2008;63(4):531‐534. doi:10.1002/ana.21324 18306241

[11] Zekeridou A , McKeon A , Lennon VA . Frequency of synaptic autoantibody accompaniments and neurological manifestations of thymoma. JAMA Neurol. 2016;73(7):853‐859. doi:10.1001/JAMANEUROL.2016.0603 27135398

[12] Honnorat J , Cartalat‐Carel S , Ricard D , et al. Onco‐neural antibodies and tumour type determine survival and neurological symptoms in paraneoplastic neurological syndromes with hu or CV2/CRMP5 antibodies. J Neurol Neurosurg Psychiatry. 2009;80(4):412‐416. doi:10.1136/JNNP.2007.138016 18931014 PMC2664637

[13] Honnorat J , Antoine JC . Paraneoplastic neurological syndromes. Orphanet J Rare Dis. 2007;2(1):22. doi:10.1186/1750-1172-2-22 17480225 PMC1868710

[14] Small M , Treilleux I , Couillault C , et al. Genetic alterations and tumor immune attack in Yo paraneoplastic cerebellar degeneration. Acta Neuropathol. 2018;135(4):569‐579. doi:10.1007/s00401-017-1802-y 29299667

[15] Ricci SB , Cerchiari U . Spontaneous regression of malignant tumors: importance of the immune system and other factors (review). Oncol Lett. 2010;1(6):941‐945. doi:10.3892/ol.2010.176 22870091 PMC3412538

[16] Mizukami M , Hanagiri T , Baba T , et al. Identification of tumor associated antigens recognized by IgG from tumor‐infiltrating B cells of lung cancer: correlation between ab titer of the patient's sera and the clinical course. Cancer Sci. 2005;96(12):882‐888. doi:10.1111/j.1349-7006.2005.00119.x 16367908 PMC11158788

[17] Villagrán‐García M , Farina A , Muñiz‐Castrillo S , et al. Revisiting anti‐Hu paraneoplastic autoimmunity: phenotypic characterization and cancer diagnosis. Brain Commun. 2023;5(5):fcad247. doi:10.1093/braincomms/fcad247 37794924 PMC10546956

[18] Wang L , Lou H , Li B , Li J , Yang YM . Paraneoplastic myelitis associated with durvalumab treatment for extensive‐stage small cell lung cancer. Invest New Drugs. 2022;40(1):151‐156. doi:10.1007/s10637-021-01154-x 34287773 PMC8763935

[19] Farina A , Birzu C , Elsensohn MH , et al. Neurological outcomes in immune checkpoint inhibitor‐related neurotoxicity. Brain Commun. 2023;5(3):fcad169. doi:10.1093/braincomms/fcad169 37389303 PMC10306160

