# Supplementary figures and images for: Predicting disability and mortality in CV2/CRMP5‐IgG associated paraneoplastic neurologic disorders
Source: Ann Clin Transl Neurol. 2024 Jan 22;11(3):710–8. doi: 10.1002/acn3.51991 (PMC10963297; doi:10.1002/acn3.51991)

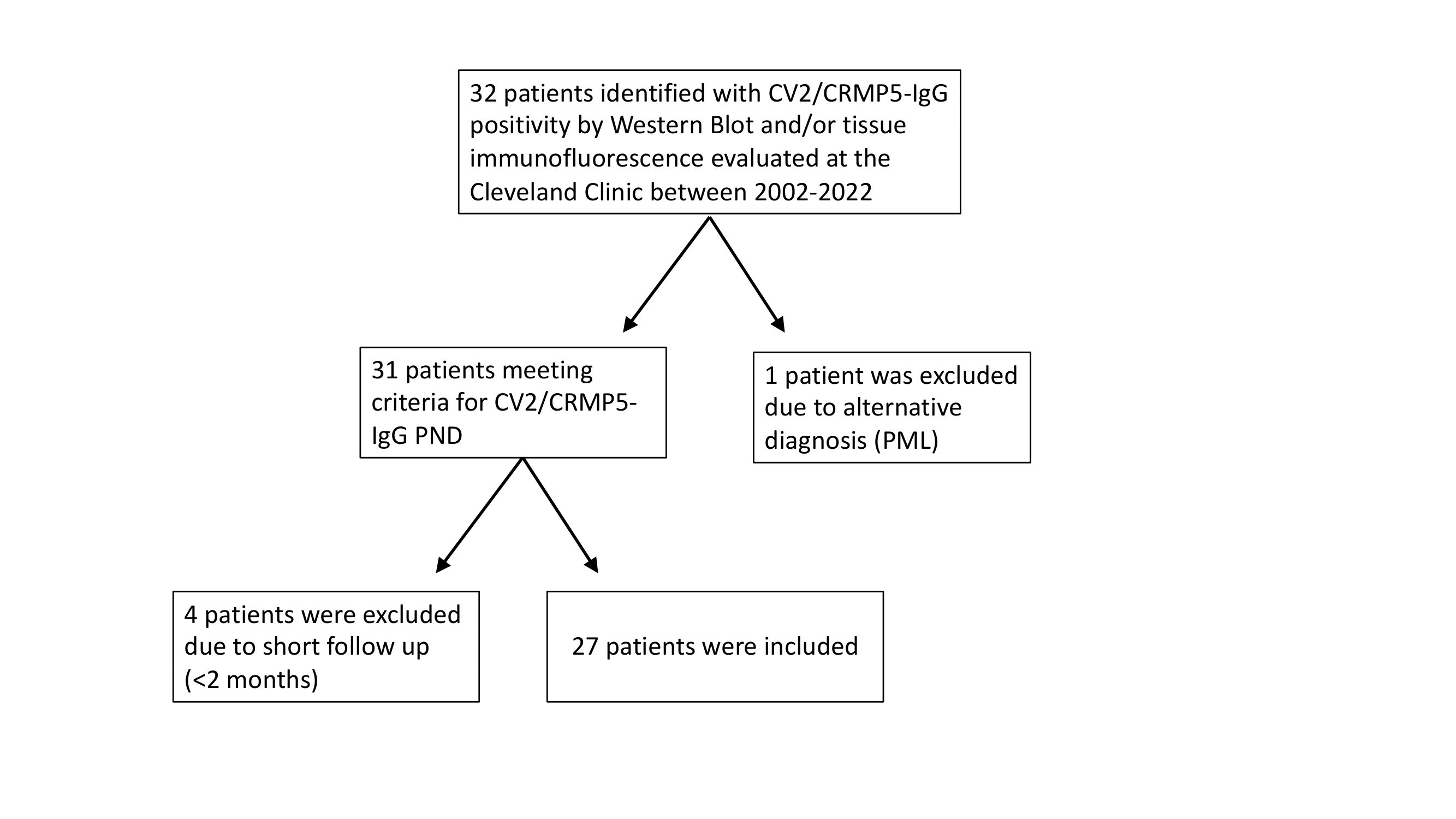

Supplement: Supplementary file 1 — Figure S1. The flow chart of inclusion criteria of the study. [file ACN3-11-710-s002.tiff]

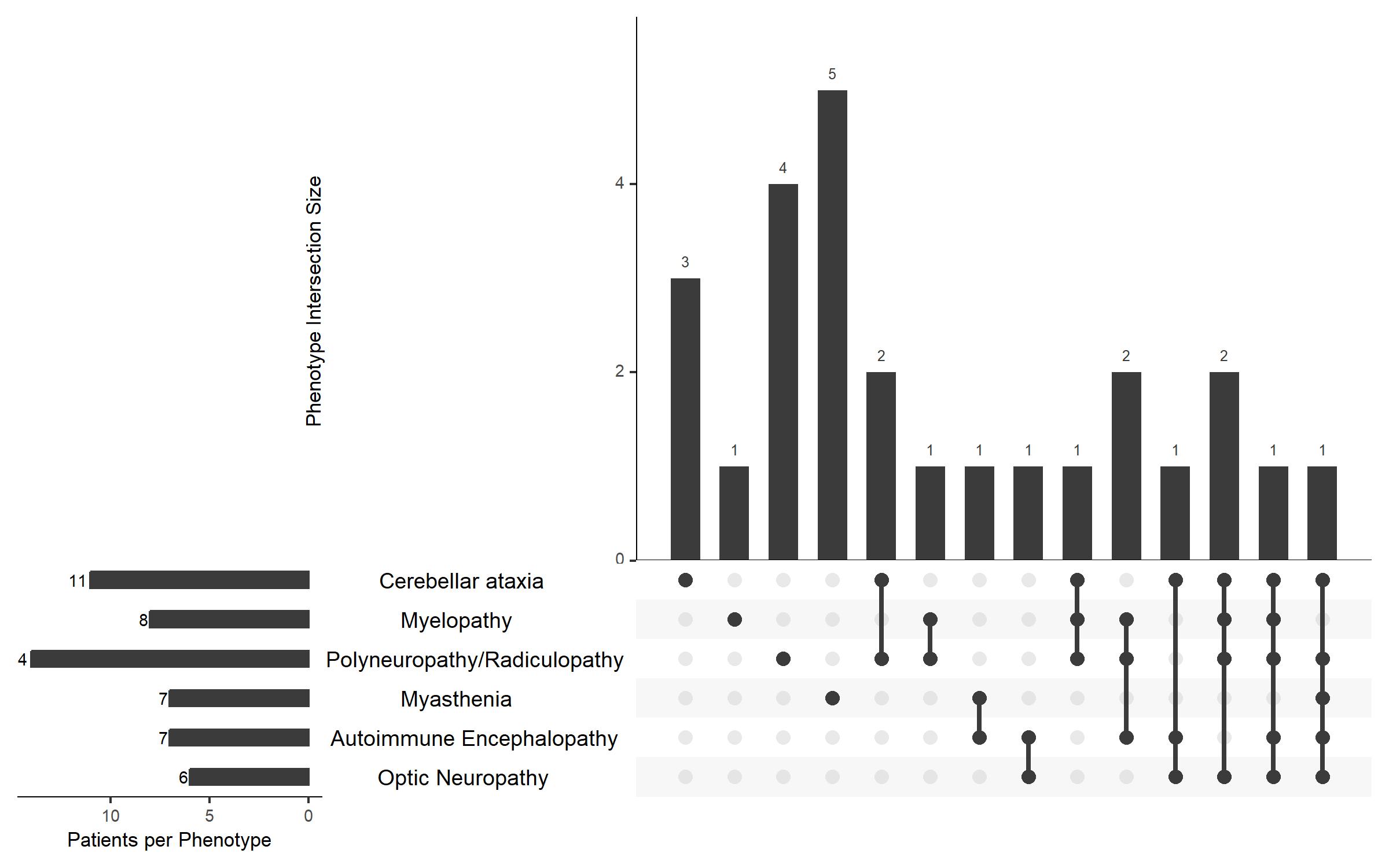

Supplement: Supplementary file 2 — Figure S2. Upset plot for the frequency of neurologic phenotype. [file ACN3-11-710-s005.tiff]

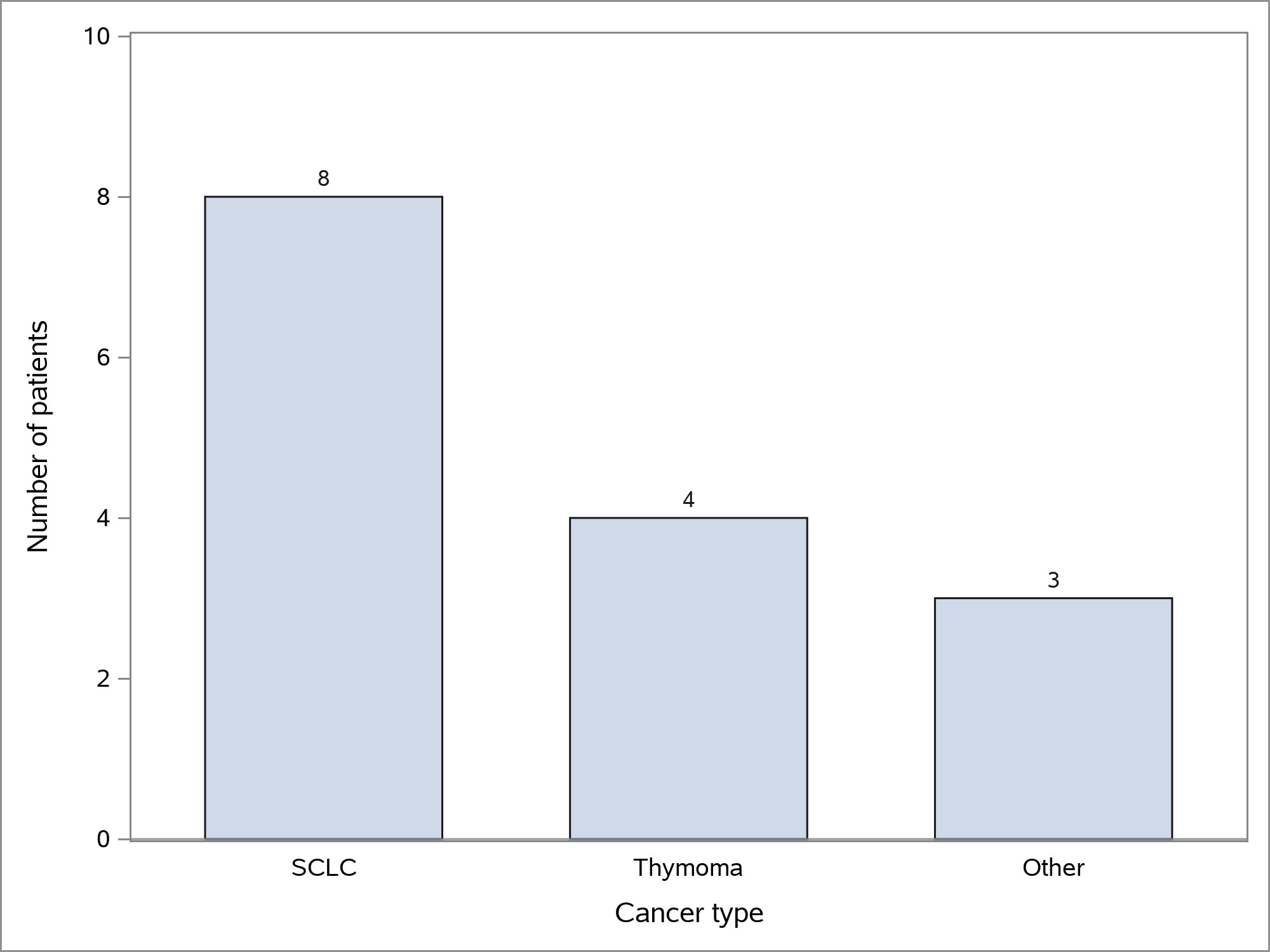

Supplement: Supplementary file 3 — Figure S3. Number of patients in each cancer type. [file ACN3-11-710-s004.tiff]

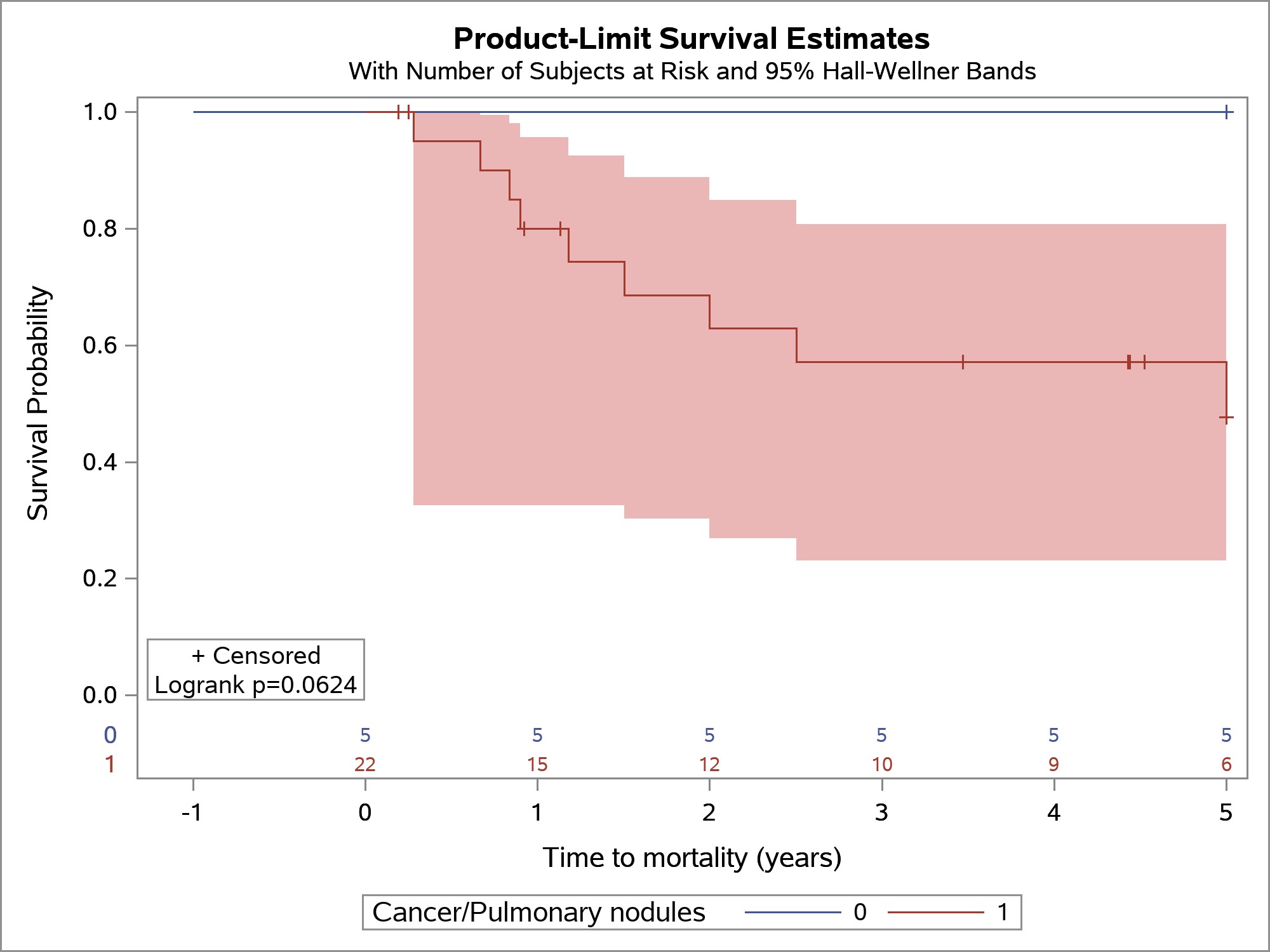

Supplement: Supplementary file 4 — Figure S4. Kaplan–Meier curve for time to mortality, stratified by cancer/pulmonary nodules vs none. [file ACN3-11-710-s006.tiff]

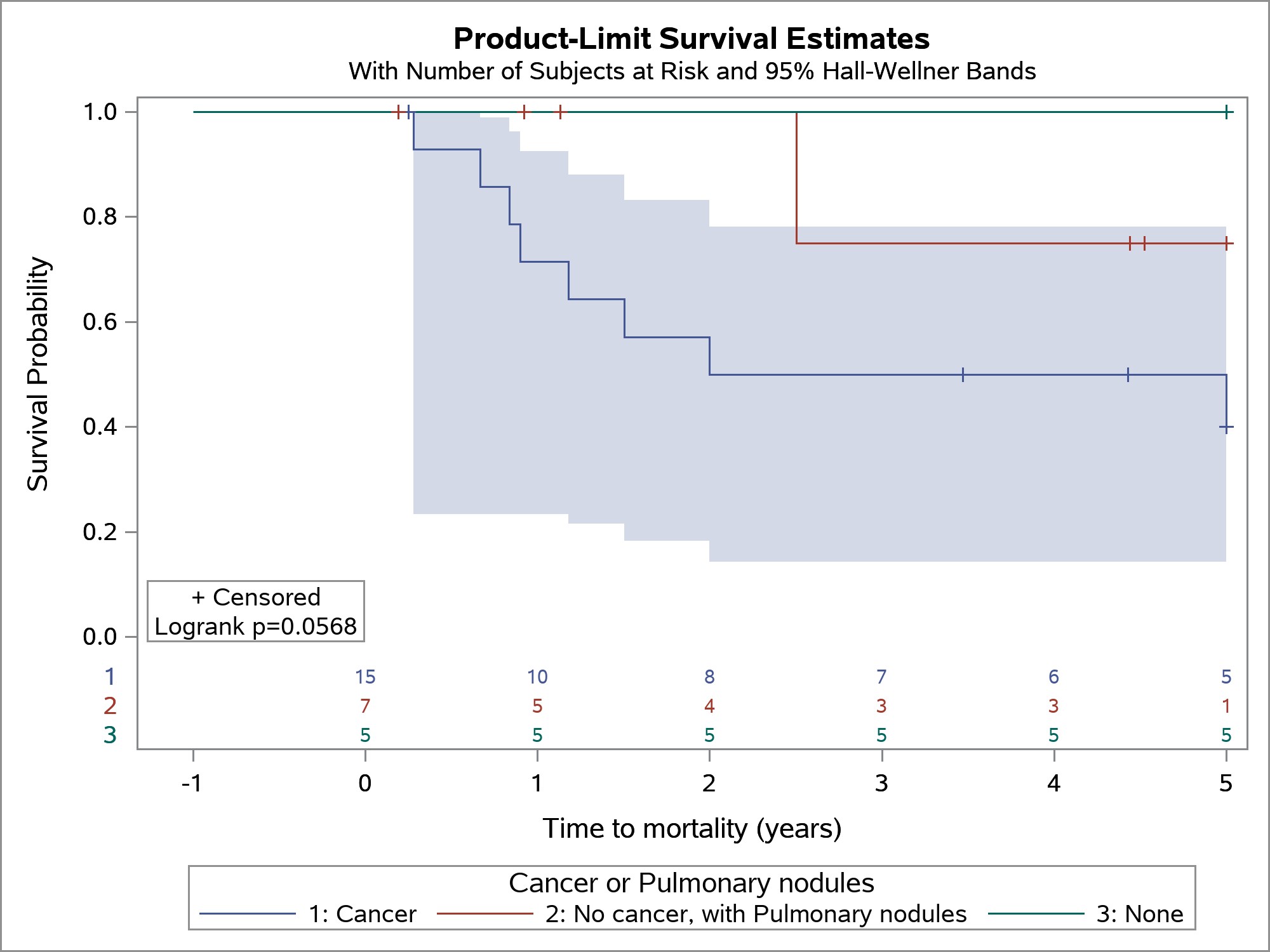

Supplement: Supplementary file 5 — Figure S5. Kaplan–Meier curve for time to mortality, stratified by cancer vs no cancer, with pulmonary nodules vs none. [file ACN3-11-710-s003.tiff]
