# Supplementary material for: Predicting disability and mortality in CV2/CRMP5‐IgG associated paraneoplastic neurologic disorders
Source: Ann Clin Transl Neurol. 2024 Jan 22;11(3):710–8. doi: 10.1002/acn3.51991 (PMC10963297; doi:10.1002/acn3.51991)
Supplement: Supplementary file 6 — Table S1. Characteristics of the study sample, stratified by phenotypes. Table S2. Comparison between patients with and without thymoma. Table S3. Comparison between patients with and without myasthenia gravis. [file ACN3-11-710-s001.docx]

**Supplementary Table 1: Characteristics of the study sample, stratified by phenotypes**

|  | Ataxia (N=11) | Myelopathy (N=8) | Polyneuropathy/  Radiculopathy (N=14) | Myasthenia (N=7) | Autoimmune Encephalopathy (N=7) | Optic Neuropathy (N=6) |
| --- | --- | --- | --- | --- | --- | --- |
| Age at symptom onset (median [Q1, Q3], years) | 66.0 [55.0, 74.0] | 69.0 [62.0, 72.5] | 66.0 [59.0, 71.0] | 56.0 [43.0, 70.0] | 71.0 [55.0, 74.0] | 71.5 [59.0, 74.0] |
| Male sex (n, %) | 6 (54.5) | 2 (25.0) | 6 (42.9) | 4 (57.1) | 3 (42.9) | 2 (33.3) |
| Pulmonary nodules in patients without cancer* (n, %) | 4 (66.7) | 2 (100.0) | 4 (66.7) | 1 (50.0) | 2 (100.0) | 2 (100.0) |
| Cancer (n, %) |  |  |  |  |  |  |
| SCLC (n, %) | 3 (60.0) | 4 (66.7) | 6 (75.0) | - | 3 (60.0) | 2 (50.0) |
| Thymoma (n, %) | - | - | - | 4 (80.0) | 1 (20.0) | - |
| Other (n, %) | 2 (40.0) | 2 (33.3) | 2 (25.0) | 1 (20.0) | 1 (20.0) | 2 (50.0) |
| Co-existent Ab (n, %) | 5 (45.0) | 3 (37.5) | 7 (50.0) | 7 (100) | 3 (42.9) | 2 (33.3) |
| AChR (binding) (n, %) | 2 (40.0) | - | 2 (28.6) | 7 (100) | 2 (66.7) | 1 (50.0) |
| ANNA1 (n, %) | 3 (60.0) | 2 (66.7) | 3 (42.9) | - | 1 (33.3) | 1 (50.0) |
| GAD65 (n, %) | - | 1 (12.5) | - | - | - |  |
| Smoking history (n, %) | 9 (81.8) | 8 (100.0) | 12 (85.7) | 3 (42.9) | 5 (71.4) | 5 (83.3) |
| CSF inflammatory findings* (n, %) | 6 (85.7) | 6 (85.7) | 7 (87.5) | - | 4 (100.0) | 4 (100.0) |
| Deceased (n, %) | 3 (27.3) | 5 (62.5) | 7 (50.0) | 3 (42.9) | 5 (71.4) | 2 (33.3) |
| Immunosuppressive therapy (n, %) | 6 (54.5) | 5 (62.5) | 7 (50.0) | 6 (85.7) | 4 (57.1) | 4 (66.7) |
| Wheelchair dependence due to PND (n, %) | 5 (45.5) | 7 (87.5) | 7 (50.0) | 0 (0.00) | 3 (42.9) | 2 (33.3) |
| mRS at first visit (median [Q1, Q3]) | 2.0 [2.0, 3.0] | 3.5 [2.5, 4.5] | 2.0 [2.0, 4.0] | 2.0 [1.0, 3.0] | 3.0 [2.0, 5.0] | 3.0 [2.0, 4.0] |
| Moderate-severe mRS (mRS 3-5)  at first visit (n, %) | 4 (36.4) | 6 (75.0) | 5 (35.7) | 2 (28.6) | 5 (71.4) | 4 (66.7) |
| mRS at last visit (median [Q1, Q3]) | 3.0 [2.0, 4.0] | 4.0 [4.0, 5.0] | 3.5 [1.00, 4.0] | 3.0 [1.0, 4.0] | 4.0 [3.0, 5.0] | 3.5 [2.0, 5.0] |
| Moderate-severe mRS (mRS 3-5)  at last visit (n, %) | 7 (63.6) | 7 (87.5) | 9 (64.3) | 4 (57.1) | 6 (85.7) | 4 (66.7) |

**Supplementary Table 2: Comparison between patients with and without thymoma**

|  | Total (N=27) | No Thymoma (N=23) | Thymoma  (N=4) | p-value |
| --- | --- | --- | --- | --- |
| Age at symptom onset (median [Q1, Q3], years) | 65.0 [54.0, 71.0] | 66.0 [58.0, 71.0] | 43.0 [42.0, 49.5] | **0.010^b^** |
| Myasthenia (n, %) | 7 (25.9) | 3 (13.0) | 4 (100.0) | **0.002^d^** |
| Deceased (n, %) | 13 (48.1) | 11 (47.8) | 2 (50.0) | 0.99^d^ |
| Moderate-severe mRS (mRS 3-5) at last visit (n, %) | 18 (66.7) | 16 (69.6) | 2 (50.0) | 0.58^d^ |
| Wheelchair dependence due to PND (n, %) | 10 (37.0) | 10 (43.5) | 0 (0.00) | 0.26^d^ |

**Supplementary Table 3: Comparison between patients with and without myasthenia gravis**

|  | **Total (N=27)** | **No Myasthenia (N=20)** | **Myasthenia (N=7)** | **p-value** |
| --- | --- | --- | --- | --- |
| Age at symptom onset, median [Q1, Q3], years | 65.0 [54.0, 71.0] | 65.0 [56.5, 71.0] | 56.0 [43.0, 70.0] | 0.28^b^ |
| Cancer, n (%) | 15 (55.6) | 10 (50.0) | 5 (71.4) | 0.41^d^ |
| SCLC, n (%) | 8 (29.6) | 8 (40.0) | 0 (0.00) | 0.068^d^ |
| Deceased, n (%) | 13 (48.1) | 10 (50.0) | 3 (42.9) | 0.99^d^ |
| Moderate-severe mRS (mRS 3-5) at last visit (n, %) | 18 (66.7) | 14 (70.0) | 4 (57.1) | 0.65^d^ |
| Wheelchair dependence due to PND, n (%) | 10 (37.0) | 10 (50.0) | 0 (0.00) | **0.026^d^** |
